# Attentional Prioritization of Complex, Naturalistic Stimuli Maintained in Working-Memory–A Dot-Probe Event-Related Potentials Study

**DOI:** 10.3389/fnhum.2022.838338

**Published:** 2022-04-29

**Authors:** Natalia Rutkowska, Łucja Doradzińska, Michał Bola

**Affiliations:** Laboratory of Brain Imaging, Nencki Institute of Experimental Biology, Polish Academy of Sciences, Warsaw, Poland

**Keywords:** attention, dot-probe, naturalistic stimuli, N2pc, working memory

## Abstract

Recent studies suggest that a stimulus actively maintained in working memory (WM) automatically captures visual attention when subsequently perceived. Such a WM-based guidance effect has been consistently observed for stimuli defined by simple features, such as color or orientation, but studies using more complex stimuli provided inconclusive results. Therefore, we investigated whether the WM-based guidance effect occurs also for naturalistic stimuli, whose identity is defined by multiple features and relations among them, specifically for faces and houses. The experiment consisted of multiple blocks in which participants (*N* = 28) either memorized or merely saw (WM or exposure condition, respectively) a template stimulus and then performed several dot-probe trials, with pairs of stimuli (template and control) presented laterally as distractors and followed by a target-asterisk. Analysis of reaction-times (RT) in the dot-probe task shows that memorized stimuli were prioritized by attention and points toward attention-hold, rather than capture, as a mechanism of attentional prioritization. Consistent with this interpretation, memorized items did not evoke a lateralized N2pc ERP component, thought to indicate attention shifts. However, in an exploratory ERP analysis we found evidence for a very early (100–200 ms post-stimulus) prioritization specific to the memorized faces, which is in line with the sensory recruitment theory of WM. In conclusion, our data provide evidence that complex stimuli are prioritized by attention when maintained in WM, and that the mechanism of such prioritization is based on a prolonged hold of spatial attention.

## Introduction

Contemporary theories of memory emphasize its role in the prospective guidance of perception and action (Nobre and Stokes, 2019). Particularly, the working memory (WM) system is currently recognized as the key component of a pro-active, top-down selection mechanism (Desimone and Duncan, 1995; Nobre and Stokes, 2019). While WM plays an important role in the volitional control of attention, it influences attentional selection also in an involuntary way. Specifically, stimuli encoded and actively maintained in visual WM automatically attract attention upon a subsequent presentation (review: Soto et al., 2008). This effect has been revealed, first, by dot-probe experiments, in which responses to probes presented at the location of the WM-maintained stimulus were faster, than to probes following an unfamiliar, control stimulus (Downing, 2000). Second, by visual search experiments, showing that search times are increased when a WM-maintained stimulus appears in the search array as a distractor (e.g., Soto et al., 2005; Olivers et al., 2006; Soto and Humphreys, 2007, 2009). Third, by eye-tracking studies indicating that eye-movements are automatically attracted by visual items matching the WM content (Hollingworth et al., 2013; Schneegans et al., 2014; Silvis and Van der Stigchel, 2014). Finally, by electrophysiological experiments revealing that the WM-maintained stimuli evoke an N2pc component, which is a classic index of covert attention shifts (Kumar et al., 2009; Carlisle and Woodman, 2013). Importantly, such a WM-based attention-capture effect is not a form of priming, as it was observed only when a stimulus was actively maintained in WM, but not when it was merely seen; and is considered automatic and involuntary, as it occurred even when detrimental to the task performance (review: Soto et al., 2008).

The automatic guidance of attention from WM has been so far demonstrated mainly with the use of simple stimuli, defined either by color or orientation (review: Soto et al., 2008). Stimuli varying on a single dimension of one basic feature are generally most effective in guiding bottom-up attention, as they can be processed pre-attentively and result in a pop-out search (Wolfe and Horowitz, 2017). Therefore, a question arises whether complex stimuli, whose identity is typically defined by multiple features and relations among them, are able to cause a similar WM-based attention guidance effect. The seminal dot-probe study by Downing (2000) revealed that images of faces, abstract geometric shapes, and line drawings of real life objects captured attention when maintained in WM. However, subsequent experiments using visual search paradigms did not replicate these findings. First, Houtkamp and Roelfsema (2006) used drawings of real-life objects as stimuli in a visual search task and found no evidence–neither in accuracy or RT scores, nor in the eye-tracking data–indicating that such complex items captured attention when held in WM. Second, Downing and Dodds (2004) also used a visual search task with complex artificial shapes as stimuli and found no interference on search performance–as indexed by accuracy and RT in response to targets–from items concurrently held in WM. Third, Peters et al. (2009) conducted an event-related potentials (ERP) study–also using complex artificial shapes as WM items–and showed not only that the memory-matching distractors did not influence the search task accuracy, but also that complex stimuli held in WM do not differ from control stimuli in terms of evoked ERP activity. Finally, a key study was conducted by Zhang et al. (2010), who used the same procedure as Peters et al. (2009) to directly compare two different sets of stimuli–one consisting of simple shapes [previously used by Soto et al. (2005)], and another of more complex, artificial shapes [used by Downing and Dodds (2004)]. Zhang et al. (2010) found that simple stimuli captured attention when maintained in WM, but complex stimuli did not cause such an effect. Therefore, they concluded that the WM-based attentional guidance critically depends on the stimulus features, with only the simple ones being effective in guiding attention from visual WM.

In light of such conflicting findings–with the classic study of Downing (2000) supporting the WM-based attention-capture by complex stimuli, but several subsequent studies challenging his conclusions (Downing and Dodds, 2004; Houtkamp and Roelfsema, 2006; Peters et al., 2009; Zhang et al., 2010)–we designed a study to provide further evidence, either in favor or against the discussed effect. Specifically, we investigated whether two types of complex, naturalistic stimuli–images of faces and houses–are prioritized by attention when maintained in visual WM. We chose these two categories as both are defined by multiple features and thought to exhibit similar levels of complexity, and thus are often compared in visual perception studies (Filliter et al., 2016). In the conducted experiment images of faces and houses were used as template stimuli, which were either memorized for later recollection (WM condition) or merely seen without the need to memorize (exposure condition), in separate blocks. Next, within each block participants performed a sequence of dot-probe trials, in which the target dot followed either a template (congruent trials) or a control image (incongruent trials), which were presented as task-irrelevant distractors (MacLeod et al., 1986). We analyzed a difference in reaction-times (RT) between congruent and incongruent trials, which is a primary index of attention capture in the dot-probe task, and compared it between experimental conditions. Further, to obtain a measure of attentional prioritization that is time-resolved and independent of behavioral response, we recorded electro-encephalographic (EEG) activity and analyzed two lateralized ERP components. First, the N2 posterior contralateral (N2pc), which is considered to represent covert attention shifts (Luck and Hillyard, 1994; Eimer, 1996; Kiss et al., 2008). N2pc is defined as more negative amplitude of signals recorded at posterior electrodes contralateral to the presented stimulus (in comparison to ipsilateral electrodes) in the early time-window (starting c.a. 200 ms after stimulus onset). Second, the Sustained Posterior Contralateral Negativity (SPCN), defined in a similar manner but occurring later, around 300–400 ms after stimulus onset, and thought to reflect maintenance and manipulation of information in visual WM (Jolicæur et al., 2008; Emrich et al., 2009; Clark et al., 2015).

We hypothesized that template stimuli will attract attention in the WM condition–as indicated by shorter RT in the congruent dot-probe trials, and by greater amplitude of the N2pc ERP component–but we did not expect to observe these effects in the mere exposure condition. Further, because we assumed the attentional prioritization of WM items to be automatic and involuntary, we did not expect to observe the SPCN ERP component, neither in the WM, nor in the exposure condition. Finally, considering preferential processing of faces in the visual system, in comparison to houses, we expected them to benefit from attentional and memory advantage (in line with: Farah et al., 1998; Tsao and Livingstone, 2008; Curby et al., 2009).

## Materials and Methods

### Participants

The study was conducted with the approval of the human ethics committee of the SWPS University of Social Sciences and Humanities (Warsaw, Poland). All participants declared normal or corrected-to-normal vision and no history of mental or neurological disorders. All participants provided written informed consent and received monetary compensation for their time (100 PLN = c.a. 25 EUR).

We analyzed data of 28 participants (18 females, mean age = 24.2, SD = 2.59 years, range: 19–28, 2 left-handed). Data of six additional participants were collected, but they were excluded from the analysis: four participants due to the technical problems during an EEG recording procedure; one participant did not comply with the dot-probe task instruction; and one participant due to insufficient number of epochs remaining after EEG signal pre-processing (detailed criteria are described in the Section “Electro-Encephalographic Recording and Analysis”).

### Stimuli

Two sets of stimuli were used. First, 60 pictures of faces with neutral expression (30 male, 30 female; all Caucasian) selected from the Karolinska Directed Emotional Faces stimulus set (KDEF; Lundqvist et al., 1998). From their original format, the face photographs were converted to grayscale using the Gnu Image Manipulation Program (GIMP^1^). Second, 60 pictures of houses from the DalHouses stimulus set (Filliter et al., 2016). All houses’ images were originally in grayscale, presented on a white background, thus no modifications were introduced. Identifiers of stimuli used in the present study can be found in the project description at OSF.^2^

### Procedure

The experimental procedure was written in the Presentation software (Neurobehavioral Systems, Albany, CA, United States) and presented on a FlexScan EV-2450 (Hakusan, Ishikawa, Japan) screen through an Intel Core i3 computer. Participants were seated comfortably in a dimly lit room with a viewing distance of 57 cm, which was maintained by a chinrest.

The procedure started with a display providing participants with the task instructions and information about the trial structure. The procedure consisted of two tasks: a working-memory (WM) task and a mere exposure task; and involved two stimuli types: faces and houses. Thus, there were four conditions: a face WM condition; a house WM condition; a face exposure condition; and a house exposure condition; which were presented to participants as separate blocks, the order of which was randomized. Each condition was further sub-divided into 32 memory or exposure blocks. For each memory/exposure block one template and one control stimulus were randomly chosen from the pool of all houses or faces. Additionally, in the face conditions the face stimuli were gender-matched, i.e., female and male template images were paired only with, respectively, female and male control images. In both, a face WM condition and a face exposure condition, female faces were used in half of the blocks, and male faces in the other half. All stimuli were presented against a black background.

**FIGURE 1:**
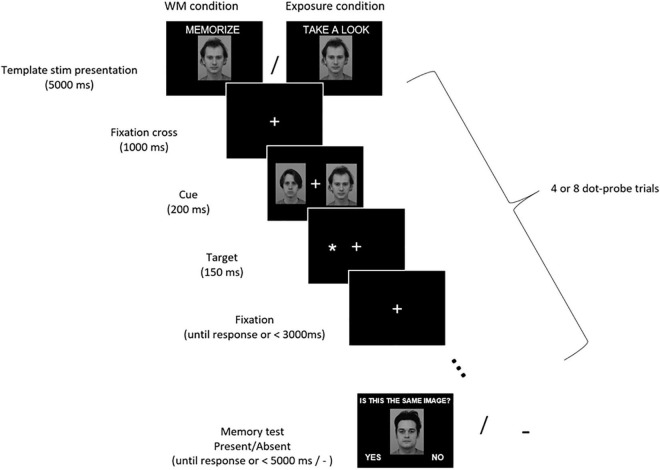
Experimental procedure. Participants were asked to memorize (WM condition) or just look (exposure condition) at the presented stimulus (either a face or a house). Then they performed 4 or 8 dot-probe trials in which their task was to indicate with a button press the location of a target asterisk (left or right). Asterisks were preceded by pairs of stimuli, which participants were supposed to ignore. A memorized/seen stimulus was presented on one side, and a control one on the other side. In the WM condition participants completed a memory test, in which they had to decide if the presented stimulus is the one they were instructed to memorize (in the exposure condition there was no memory test). Images from KDEF stimulus set reproduced with permission from Lundqvist et al. (1998). IDs of images included in the Figure: AM13NES and AM22NES.

Each of those 32 blocks started with a central presentation of a template stimulus for 5000 ms (Figure 1). The instruction–either “Memorize this picture” (in the WM condition) or “Take a look at this picture” (in the exposure condition)–was displayed above the image. The face images subtended 7.4° × 10.0° of the visual angle, while house images varied in size and subtended from 6.1° to 9.3° × 7.8° of the visual angle. After the display of the template stimulus, a sequence of dot-probe trials was presented. Each dot-probe trial started with a fixation cross (subtending 0.9° × 0.9° of the visual angle) displayed in the center of the screen. The fixation cross remained on-screen throughout the trial. After 1000 ms a pair of stimuli were presented bilaterally for 200 ms–the template stimulus on one side and control stimulus on the other. Face stimuli were presented with their inner edge 4.4° left and right from the fixation cross, while house stimuli with the inner edge from 3.8° to 5.3° left and right from the fixation cross. Next, a target asterisk subtending 0.7° × 0.7° of the visual angle was presented for 150 ms in the location of the center of either the template stimulus (congruent trial) or the control one (incongruent trial). Participants were instructed to maintain their gaze on the centrally presented fixation cross, ignore the laterally appearing stimuli, and indicate the side of the target asterisk presentation (left or right) by pressing one of two buttons using index fingers of their left or right hand. Participants were asked to respond as quickly and accurately as possible. The response time to the target asterisk was limited to 3000 ms and the next trial started immediately after the manual response. Within each dot-probe sequence the template stimulus was presented on the left side in half of the dot-probe trials, and on the right side in the other half. Further, half of the dot-probe trials were congruent and half were incongruent. The order of trials within each sequence was randomized.

In the WM condition, participants were given a memory test at the end of each memory block (i.e., after completing the dot-probe trials sequence). Either a template image or a different image (neither a template, nor a control stimulus) was presented centrally. Participants had to indicate whether the presented stimulus is the same or different from the one they were previously asked to memorize. Above the image a question “Is this the same image?” was displayed. In half of the WM blocks the stimulus was the template image (correct answer “yes”), and in the other half it was a different image (correct answer “no”). The answer “yes” was displayed in the left corner of the screen and the answer “no” in the right corner. Participants responded by pressing one of two buttons (left for “yes,” right for “no”). The response time in the memory test was limited to 5000 ms and the next WM block started immediately after the manual response. In the mere exposure condition participants were not tested for stimulus recognition, but immediately after completion of the dot-probe sequence the next exposure block started.

In total 192 dot-probe trials were presented per condition. Within each condition half of the WM/exposure blocks comprised 4 dot-probe trials, and the other half comprised 8 dot-probe trials (the order of blocks was random). The number of trials varied in order to prevent participants from expecting the exact moment of a memory test, and thus encourage them to maintain the WM active throughout the block. Participants had a self-paced break five times per condition.

### Analysis of Behavioral Data

All analyses of behavioral data were conducted using custom-made Python scripts. Accuracy of responses to the presentation side of the target-dot was calculated as a percentage of correct responses. The obtained values are presented in the Results section, but due to ceiling level performance in the majority of participants this measure was not analyzed statistically. Therefore, analysis of the dot-probe task data was focused on establishing whether reaction times (RT) of manual responses to the target asterisk differ between two types of trials: those in which the asterisk was presented on the same side as the potentially attention-grabbing stimulus (memorized/seen face or house; congruent trials) and those in which the asterisk was presented on the neutral stimulus side (control face or house; incongruent trials). Mean reaction times were calculated only for the correct responses. For the WM condition the accuracy of memorizing a template stimulus was calculated as a percentage of correct responses in the memory test.

### Electro-Encephalographic Recording and Analysis

During the experiment, EEG signal was recorded with 64 Ag-AgCl electrically shielded electrodes mounted on an elastic cap (ActiCAP, Munich, Germany) and positioned according to the extended 10–20 system. Vertical (VEOG) and horizontal (HEOG) electro-oculograms were recorded using bipolar electrodes placed at the supra- and sub-orbit of the right eye and at the external canthi. Electrode impedances were kept below 10 kΩ. The data were amplified using a 128-channel amplifier (QuickAmp, Brain Products, Enschede, Netherlands) and digitized with BrainVisionRecorder^®^ software (Brain Products, Munich, Germany) at a 500 Hz sampling rate. The EEG signal was recorded against an average of all channels calculated by the amplifier hardware.

Electro-encephalographic and EOG data were analyzed using EEGlab 14 functions and Matlab 2016b. First, all signals were filtered using a high-pass (0.5 Hz) and a low-pass (45 Hz) Butterworth IIR filter (filter order = 2; Matlab functions: *butter* and *filtfilt*). Then data were re-referenced to the average of signals recorded from left and right earlobes, and down-sampled to 250 Hz. All data were divided into 768 dot-probe epochs (192 epochs per condition; [−200, 1200] ms with respect to the faces/houses images onset) and the epochs were baseline-corrected by subtracting the mean of the pre-stimulus period (i.e., [−200, 0 ms]). Further, epochs were rejected based on the following criteria (all values Mean ± SEM): (i) when there was no manual response to the target dots until 1.2 s after the onset (18.9 ± 6.0; range [0, 120] epochs per participant); (ii) when activity of the HEOG electrode in the time-window [−200, 500] ms exceeded −40 or 40 uV (105.5 ± 19.1; range [11, 352] epochs per participant); (iii) when activity of the P7 or P8 electrode in the time-window [−200, 600] ms exceeded −80 or 80 uV (none of the epochs rejected). Thus, after applying the described criteria the average number of analyzed epochs per participant was: 643.6 ± 22.0; range [355, 752].

A participant was excluded if the number of epochs in any condition was <60. This criterion resulted in excluding 1 participant out of 29 (but additional 5 participants were excluded due to other criteria, as described in the Section “Participants”). The numbers of epochs provided above were calculated based on the final sample of 28 participants.

Next, each EEG-EOG data-set was decomposed into 50 components using Independent Component Analysis as implemented in the EEGlab *pop_runica* function. To remove residual oculographic artifacts from the data the following procedure was used: time-course of each component was correlated with time-courses of HEOG and VEOG electrodes and in case the Spearman correlation coefficient exceeded −0.3 or 0.3 a component was subtracted from the data. Using this procedure 3.0 ± 0.2 components (range [1, 6]) per participant were removed.

To ensure that there was no difference in the number of trials with the target presented on the WM (congruent) and control side (incongruent) which were retained in the data after preprocessing, we conducted a 3-way repeated-measures ANOVA with *congruency* (congruent, incongruent trials), *task* (WM, exposure), and *stimulus* (face, house) as factors. We confirmed that *congruency* had neither main effect [*F*_(1,27)_ = 1.386, *p* = 0.249, η_*p*_^2^ = 0.049], nor interacted with other factors [*congruency* × *task*: *F*_(1,27)_ = 0.649, *p* = 0.428, η_*p*_^2^ = 0.023, *congruency* × *stimulus*: *F*_(1,27)_ = 0.042, *p* = 0.839, η_*p*_^2^ = 0.002].

Both N2pc and SPCN components are defined as a difference between the contralateral and ipsilateral activity recorded at posterior electrodes after a stimulus presentation (Luck and Hillyard, 1994; Jolicæur et al., 2008). As N2pc is typically maximal at posterior electrodes sites (Luck, 2012), signals from P8 and P7 electrodes were used to calculate both components, similarly to other studies using N2pc and SPCN as markers of attention shifts (Kappenman et al., 2014; Furtak et al., 2020; Bola et al., 2021). Epochs were divided with respect to the condition and presentation side of the template stimulus in the following way: when template stimulus was presented on the left side, P8 was the contralateral electrode and P7 was the ipsilateral electrode; when template stimulus was presented on the right side, P7 was the contralateral electrode and P8 was the ipsilateral electrode. For each condition contralateral and ipsilateral signals were first concatenated and then averaged, to obtain contralateral and ipsilateral waveforms. These waveforms were averaged within the 200–400 ms time-window for the N2pc analysis (e.g., Woodman et al., 2009; Reutter et al., 2017; Wójcik et al., 2019) and in the 400–600 ms time-window for the SPCN analysis (Clark et al., 2015). Further, based on the visual inspection of the obtained ERP waveforms (Figure 3) an exploratory analysis was conducted on lateralized activity observed in the early, 100–200 ms time-window.

### Statistical Analysis

Statistical analyses were conducted in the JASP software and cross-checked with Statcheck.^3^ The values are reported as Mean ± SEM, unless stated otherwise. For all statistical tests probability values were reported (*p*) and the standard 0.05 alpha level was used as a threshold for refuting the null hypothesis.

To test for the presence of the behavioral (RT) and electrophysiological dot-probe task effects repeated–measures (rm) ANOVA models were used. Specific to the analysis of RT was the factor of *congruency*, defined by the asterisk presentation side with respect to the memorized/seen item (congruent vs. incongruent trials). Specific to the electrophysiological analysis was the factor of *side*, defined as the side on which ERP activity was recorded, with respect to the memorized/seen item (ipsi- vs. contra-lateral). The *side* effect was analyzed separately for activity recorded in the three analyzed time-windows (100–200 ms, 200–400 ms, and 400–600 ms). The factors of a *stimulus* (faces vs. houses) and *task* (memory vs. mere exposure) were included in all models. The simple main effects analyses were conducted in case of a significant interaction. Results were reported as *F*(df) and partial eta-squared, the indicator of the effect size, was reported as η_*p*_^2^.

To compare the accuracy scores in the memory test between faces and houses conditions, the data distribution was first tested with the Shapiro–Wilk test and, as it deviated from normality, a non-parametric two-tailed Wilcoxon test was used. The statistic was reported as a sum of positive ranks (*W*), together with the matched rank biserial correlation (*r*_*rb*_) as a measure of the effect size.

### Data Availability

The data used in the statistical analysis can be accessed from the OSF (see text footnote 2). Raw EEG data, and scripts used for presentation of the experimental procedure and data analysis will be shared by authors per request.

## Results

### Memory Accuracy

We observed high working-memory (WM) accuracy scores for both faces (97.2 ± 0.7%) and houses (94.2 ± 1.6%), which indicates that participants were actively maintaining the template stimulus in working memory. Comparing the WM accuracy between face and houses we found better memory performance for face images (*W* = 172.500, *p* = 0.010, *r_*rb*_* = −0.150). This result confirms our hypothesis and is in line with previous studies (Curby et al., 2009), but due to the ceiling level performance and low effect size it should be treated with caution.

### Dot-Probe Task–Behavioral Results

**FIGURE 2:**
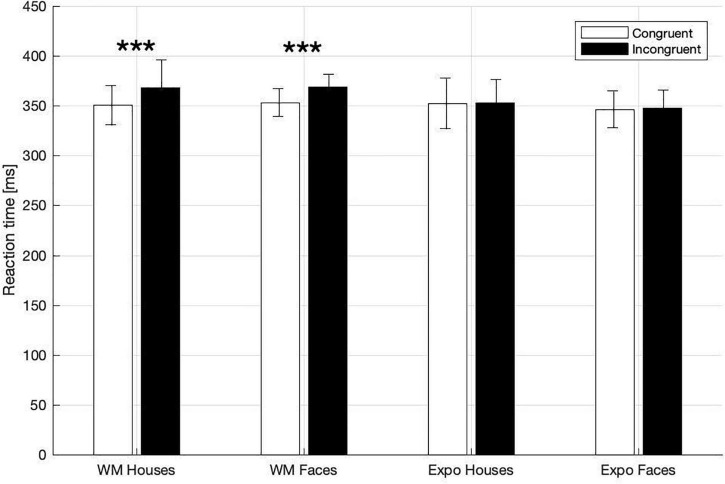
Mean RTs in dot-probe task for the WM and exposure conditions and both types of stimuli (House, Face). Asterisks indicate statistically significant differences between congruent and incongruent trials (*p* < 0.001). Error bars represent 2SEM.

In the dot-probe task participants exhibited ceiling-level accuracy (i.e., in indicating the target-dot presentation side), with the percentage of correct responses being: 97 ± 0.6% in the house WM condition; 97 ± 0.6% in the face WM condition; 97 ± 0.8% in the house exposure condition; 98 ± 0.5% in the face exposure condition. Correct responses to the target asterisk were analyzed in terms of reaction times (RT), which was our primary index of attention capture. Specifically, we investigated whether RT were shorter when the target followed a potentially attention-grabbing template stimulus (i.e., congruent trials), in comparison to trials when it followed a control stimulus (i.e., incongruent trials; Figure 2). In a three-way rm-ANOVA analysis we found a significant main effect of *congruency* [*F*_(1,27)_ = 15.49, *p* < 0.001, η_*p*_^2^ = 0.365] and *task* [WM vs. exposure; *F*_(1,27)_ = 9.05, *p* = 0.006, η_*p*_^2^ = 0.251], and a significant interaction between those two factors [*F*_(1,27)_ = 14.24, *p* = 0.001, η_*p*_^2^ = 0.345]. With regard to this interaction there was a significant simple main effect of *congruency* in the memory condition [congruent trials: 352.06 ± 8.67 ms; incongruent 368.54 ± 10.86 ms; *F*_(1,27)_ = 21.42, *p* < 0.001, η_*p*_^2^ = 0.442], but not in the exposure condition [congruent trials: 349.33 ± 11.14 ms; incongruent 350.55 ± 10.56 ms; *F*_(1,27)_ = 0.265, *p* = 0.611, η_*p*_^2^ = 0.010]. The main effect of *stimulus* [*F*_(1,27)_ = 0.052, *p* = 0.822, η_*p*_^2^ = 0.002] and other interactions [*task* × *stimulus*: *F*_(1,27)_ = 0.830, *p* = 0.370, η_*p*_^2^ = 0.03, *stimulus* × *congruency: F*_(1,27)_ = 0.033, *p* = 0.858, η_*p*_^2^ = 0.001, *congruency* × *task* × *stimulus: F*_(1,27)_ = 0.12, *p* = 0.746, η_*p*_^2^ = 0.004] did not reach significance. Therefore, in line with our hypothesis, we found attentional prioritization of stimuli that were actively maintained in WM (but not of stimuli that were merely seen), and such a WM-based effect was observed irrespective of the stimulus type.

### Dot-Probe Task–Electrophysiological Results

**FIGURE 3:**
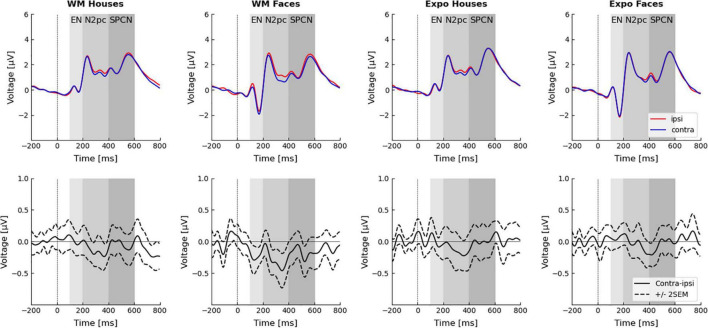
Event related potentials in the dot-probe task. Electrodes P7/P8 were chosen for the analysis. Waveforms recorded ipsi- and contra-laterally with respect to the seen or memorized stimulus are presented in the top row. Difference waveforms (i.e., contra–ipsi-lateral side) are presented in the bottom-row. Time windows of the three analyzed components–the early negativity (EN; 100–200 ms), N2pc (200–400 ms), and SPCN (400–600 ms)–are highlighted.

Results of the three-way rm-ANOVA for the N2pc ERP component indicate a significant *side* effect (ipsilateral amplitude: 1.64 ± 0.44 μV; contralateral: 1.49 ± 0.44 μV), but interactions between *side* and *task*, or *side* and *stimulus* were not significant (Figure 3 and Table 1). Therefore, we conclude that our manipulations did not affect the N2pc component, and that our hypothesis stating that larger N2pc will be observed in the WM task was not confirmed.

**TABLE 1:** Rm-ANOVA analysis of the electrophysiological effects.

	Time window: 100–200 ms			Time window: 200–400 ms			Time window: 400–600 ms		
Factor	*F*(1,27)	*p*	η_*p*_^2^	*F*(1,27)	*p*	η_*p*_^2^	*F*(1,27)	*p*	η_*p*_^2^
Side	1.556	0.223	0.054	10.194	0.004	0.274	1.717	0.201	0.060
Task	0.661	0.423	0.024	0.032	0.860	0.001	0.587	0.450	0.021
Stimulus	31.910	<0.001	0.542	1.023	0.321	0.037	10.061	0.004	0.271
Side × Task	4.923	0.035	0.154	1.899	0.180	0.066	1.414	0.245	0.050
Side × Stim	0.066	0.321	0.036	0.069	0.795	0.003	0.672	0.420	0.024
Task × Stim	2.341	0.138	0.080	0.027	0.871	0.001	0.394	0.536	0.014
Side × Task × Stim	4.923	0.035	0.154	2.032	0.165	0.070	0.224	0.640	0.008

*Analysis was conducted separately for three time windows (early negativity: 100–200 ms; N2pc: 200–400 ms; and SPCN: 400–600 ms). Each model included the following three factors: side (recording from contralateral/ipsilateral electrodes), task (memory/exposure), stimulus (face/house).*

Similarly, results of the three-way rm-ANOVA for the SPCN ERP component indicate a significant *stimulus* effect only. This effect indicates that activity evoked by faces (1.77 ± 0.37 μV) had lower amplitude than activity evoked by houses (2.15 ± 0.33 μV). However, because none of the interactions involving the *side* factor was significant, we conclude that our manipulation did not affect the SPCN component, which is in line with our hypothesis.

Inspection of the obtained ERP waveforms prompted us to conduct an unplanned, exploratory analysis of the lateralized activity in an earlier, 100–200 ms time-window. This analysis yielded a significant interaction between *side*, *task*, and *stimulus* (Figure 3 and Table 1). The simple main effects analysis showed that an early contralateral negativity is present in the memory condition for faces [ipsilateral: −0.51 ± 0.39 μV; contralateral: −0.74 ± 0.38 μV; *F*_(1,27)_ = 10.13, *p* = 0.004, η_*p*_^2^ = 0.273], but not for houses [ipsilateral: 0.15 ± 0.43 μV; contralateral: 0.15 ± 0.44 μV; *F*_(1,27)_ = 0.004, *p* = 0.951, η_*p*_^2^ < 0.001]. In the mere exposure conditions the simple main effects were not significant neither for faces [ipsilateral: −0.85 ± 0.42 μV; contralateral: −0.79 ± 0.41 μV; *F*_(1,27)_ = 1.02, *p* = 0.322, η_*p*_^2^ = 0.036] nor for houses [ipsilateral: 0.23 ± 0.42 μV; contralateral: 0.23 ± 0.43 μV; *F*_(1,27)_ = 0.01, *p* = 0.971, η_*p*_^2^ < 0.001]. Therefore, our analysis revealed that the very early lateralized activity was evoked only by face images maintained in WM.

## Discussion

The present study examined whether complex naturalistic stimuli that are actively maintained in visual WM are automatically prioritized by attention upon subsequent presentation. Such a WM-guided attentional selection has been consistently observed for simple stimuli (review: Soto et al., 2008), but inconsistent findings were reported when more complex stimuli were used (Downing, 2000; Downing and Dodds, 2004; Houtkamp and Roelfsema, 2006; Peters et al., 2009; Zhang et al., 2010). Therefore, in the present study images of faces and houses were either memorized or merely seen by participants, and subsequently presented in a dot-probe task. To test a hypothesis that such complex, naturalistic stimuli are automatically prioritized by attention when actively maintained in WM we analyzed both behavioral (RT) and electrophysiological (N2pc) indexes of attentional prioritization.

### Attentional Prioritization of Complex Stimuli

Our main finding from the dot-probe task is that RTs were significantly shorter when a target asterisk followed a memorized face or a house, in comparison to the situation when it followed a control stimulus. Importantly, the RT effect was not observed, neither for faces nor for houses, when participants merely saw the template images, without an instruction to memorize them. This indicates that stimuli actively maintained in WM were prioritized by attention, but those that were merely seen did not cause such an effect. Thus, our behavioral results replicate the findings of Downing (2000), who found a similar effect for a range of complex stimuli–including complex artificial shapes, line drawings, and images of faces–in a similar dot-probe task. However, his findings were challenged by subsequent visual search experiments, which used drawings of real-life objects and complex shapes and did not find a similar effect (Downing and Dodds, 2004; Houtkamp and Roelfsema, 2006; Peters et al., 2009). Of special relevance is work by Zhang et al. (2010), who compared two sets of artificial shapes–simple and complex ones–directly in the same study and using the same visual search task. They found only the simple stimuli to be prioritized by attention when maintained in WM and concluded that the attentional guidance critically depends on the stimulus features, with a stronger effect for simple than for complex stimuli. Thus, their conclusion is at odds with results of both Downing (2000) and our study, which show that even stimuli defined by multiple features and by relations among them can induce a WM-based attentional bias. Importantly, our data provide evidence that the mere complexity of a stimulus is not likely to be a critical factor in the investigated phenomenon. We rather argue that the images used in our study evoked the WM-based guidance effect because they were processed holistically and perceived as meaningful, in contrast to the complex artificial shapes [e.g., used by Zhang et al. (2010)]. Our interpretation is in line with Xu (2017), arguing that visual WM is not typically used to encode features of a single dimension, but rather to store integrated representations of meaningful objects.

Notably, apart from differences in stimuli properties, the discussed studies differ also in terms of paradigms used–while the studies demonstrating a WM-based prioritization of complex stimuli used a dot-probe task (Downing, 2000, and our study), studies finding no evidence for such an effect used a visual search task (Downing and Dodds, 2004; Houtkamp and Roelfsema, 2006; Peters et al., 2009; Zhang et al., 2010). Potential differences in sensitivity between these two procedures might thus account, at least partially, for differences in reported results. More data has to be collected by future studies to address which factor plays a key role in the discussed effect.

Two opposing accounts of the mechanism behind the WM-based guidance effect have been proposed. First one emphasizes the role of verbal (and perhaps semantic) representations in WM maintenance and subsequent directing of visual attention. It is based on studies showing that verbalization by itself can induce attention guidance (Soto and Humphreys, 2007), and that when visual stimuli are used as memory items the articular suppression task impairs the guidance effect (Downing and Dodds, 2004; Woodman and Luck, 2007; Soto and Humphreys, 2008). In contrast, the second view assumes that the guidance effect relies predominantly on visual representations. It is supported by experiments revealing the effect only when stimuli were defined by small and hard to verbalize differences in their attributes (e.g., hues of one color or slightly differing shapes) but not when easy to verbalize categorical differences were used (Olivers et al., 2006). Importantly, the stimuli used in our study were also difficult to verbalize and required maintaining a predominantly visual representation. Thus, our results provide further support for the latter view.

### Attentional Prioritization–Capture or Hold?

The majority of previous studies investigated the WM-based guidance effect using behavioral methods, and thus relevant EEG or fMRI data is scarce. Therefore, in the present study we collected EEG data, with the main aim of using them to evaluate the time-course of attentional prioritization. However, the N2pc ERP component–a classic index of attention capture (Luck and Hillyard, 1994; Eimer, 1996; Kiss et al., 2008)–was not affected by the memory manipulation. Thus, in our study we found a robust behavioral (RT) effect of attentional prioritization, but at the same time no related electrophysiological effect in the form of N2pc. Lack of N2pc is thus at odds with previous studies that found N2pc in similar WM tasks (Kumar et al., 2009; Carlisle and Woodman, 2013), albeit it is important to emphasize these previous studies used simple stimuli. Importantly, the series of previous studies have shown that N2pc can be observed when stimuli maintained in WM are subsequently presented as task-irrelevant distractors [Kumar et al., 2009; but see also Carlisle and Woodman (2011)], but its amplitude is four times higher when stimuli constitute task-relevant targets (Carlisle and Woodman, 2013). Thus, while absence of the N2pc component in our study might be related to the fact that complex stimuli were used, it might also stem from task-irrelevance of the memory items. Future studies will investigate the effect of naturalistic stimuli in situations when they are task-relevant, which would also more closely reflect daily life situations.

Importantly, the classic theory of attention proposed by Posner et al. (1987) differentiates two independent functionalities of spatial attention orienting–attention shifts (or capture) defined as movement of attention from its current location to a new one; and attention engagement (or hold) described as an involvement in processing of a stimulus and a transient inability to switch to a new location. Therefore, another potential explanation of the dissociation between RT and N2pc observed in our study is that the memorized stimuli did not automatically capture attention (thus no N2pc effect was observed), but rather held and engaged attention for a longer time. What further supports this interpretation is that we observed an elongation of RT in the incongruent trials, rather than shortening of RT in the congruent ones–this is evident when the WM and exposure conditions are compared (Figure 2). Previous visual search studies using simple stimuli and including valid, neutral, and invalid conditions have provided conflicting results on the matter of capture vs. hold. Some found longer RTs in invalid as relative to neutral trials, but no difference between valid and neutral conditions (which would be indicative of an attention-hold by memorized items; Soto et al., 2007). However, others show both shorter RT in valid and longer RT in invalid trials, in comparison to the neutral ones (which would be indicative of both capture and hold; Soto et al., 2006). Importantly, data collected by Downing (2000) do not speak to the matter at hand because of the between-subject design used in his study (the group performing the control task had in general significantly longer RTs to probes than the group performing WM task). Thus, further studies are required to elucidate the precise mechanism of attentional prioritization of the WM-maintained items.

### Early Prioritization Specific to Faces

Even though our WM manipulation did not influence the N2pc or SPCN components, in an exploratory analysis we did find electrophysiological evidence suggesting a very early prioritization of the memorized faces. Specifically, we observed a contralateral negativity in response to the memorized face already between 100 and 200 ms after the stimulus onset. Thus, it is not clear whether contralateral negativity occurring so early can be termed N2pc, as N2pc is considered to occur around 175–200 ms after the stimulus onset (i.e., co-occurring with N2; e.g., Woodman et al., 2009; Reutter et al., 2017; Wójcik et al., 2019). Nevertheless, observing contralateral negativity already around 100 ms after the stimulus onset (i.e., co-occurring with the P100 component) suggests it represents the early and perceptual stages of processing. Enhanced activity of the occipital area in response to a stimulus held in WM has been already reported (Tan et al., 2014, 2015), but while Tan and colleagues analyzed the P100 amplitude, here we analyzed a difference between contralateral and ipsilateral activity (i.e., in our study the WM-maintained stimulus was presented always in pair with control stimulus, thus analysis of P100 is not possible). However, other studies, which used simple stimuli, did not observe any evidence for such an early WM-associated activity (e.g., Kumar et al., 2009; Telling et al., 2010). It is thus important to emphasize that in our study the early effect was present for faces, but not for houses. While the mechanisms of such an early electrophysiological effect remain to be investigated, the fact that it was observed only for faces is in line with several lines of evidence. First, due to their evolutionary and social importance, faces are processed in a largely automatic and holistic manner (Farah et al., 1998; Tsao and Livingstone, 2008). Second, due to holistic encoding strategies, faces benefit from a WM advantage (which is observed also in our data; Curby et al., 2009). Third, continuous flash suppression (CSF) studies show that faces actively maintained in WM break the CFS faster than faces that were merely seen (Pan et al., 2014). The fact that in the CFS paradigm WM can bias face perception outside of awareness is in line with the automatic and involuntary (possibly pre-attentive) nature of the effect found here. Thus, the attention prioritization revealed in the present study is a plausible mechanism accounting for the CFS effects. Finally, face recognition is performed by a specialized set of brain regions (Haxby et al., 2000; Kanwisher and Yovel, 2006) with the initial stages of face categorization occurring as early as 80–150 ms post-stimulus (Herrmann et al., 2005), which is in line with the observed early effect.

The presence of such early visual cortex activity in response to the WM-maintained stimuli is relevant to the ongoing debate on neuronal mechanisms of visual working memory. Here, two opposing theories have been proposed: first, the top-down amplification hypothesis, which assumes that visual WM items are maintained by fronto-parietal interactions (Bettencourt and Xu, 2016; Riley and Constantinidis, 2016; Christophel et al., 2018; Thigpen et al., 2019; review: Xu, 2017); second, the sensory-recruitment hypothesis, assuming that visual WM items are stored and maintained in the visual cortex (i.e., that perception and visual WM share the same neural substrate; Postle, 2006). The latter view might particularly effectively account for the automatic interactions between perception and the WM contents, which were observed in our and other studies (e.g., Silvanto and Cattaneo, 2010; Albers et al., 2013; Gayet et al., 2017; Teng and Kravitz, 2019). Importantly, given that ERP components observed in the 100–200 ms time-range are generated by sensory brain regions and reflect perceptual processing (Nusslock, 2016), such an early prioritization of the memorized faces provides support for the sensory recruitment theory. Further, such an early effect was not found in previous dot-probe studies using very salient and relevant emotional faces (Holmes et al., 2009) or self-faces of participants (Wójcik et al., 2019; Bola et al., 2021), which further indicates it might specifically reflect a match between the WM-maintained representation and an incoming stimulus. However, because this analysis was exploratory, the conclusion should be treated with caution.

### Limitations and Conclusion

There are two main limitations of our study that should be pointed out. First, we are not able to definitely exclude the possibility of strategic allocation of attention to the WM-maintained stimuli. In such a scenario participants would focus on the template image in order to improve (refresh) its representation in WM and perform better in the subsequent memory test. However, the design of our study–including brief presentation time of distractor stimuli (200 ms), the minimal demands on WM for the memory test (i.e., participants performance exhibited a ceiling-level), and the fact that occurrence of the observed WM-based attention capture effect was detrimental to the dot-probe task performance–discouraged such strategic and volitional effects. Further, considering that robust N2pc is observed when task-relevant stimuli are attended strategically (Kumar et al., 2009), and that SPCN’s amplitude increases during retention and processing of stimuli in the WM (Jolicæur et al., 2008; Emrich et al., 2009; Clark et al., 2015), the fact that neither component was observed in our data is also an argument against the strategic resampling. Second limitation is that WM and exposure conditions differed in terms of cognitive effort necessary to encode and maintain the presented stimulus. As this might be important for disentangling the exact mechanisms behind attentional effects, future studies should use the retro-cueing procedure (e.g., Gayet et al., 2017; Gayet and Peelen, 2019).

In conclusion, our study provides evidence that attentional prioritization of WM-maintained stimuli can be observed for complex and naturalistic stimuli, and thus encourages further investigations of this effect in more ecological conditions. The pattern of RT results and the dissociation between RT and N2pc suggests that the observed attentional prioritization might reflect hold, rather than capture of attention. Further, our electrophysiological results provide evidence for a very early prioritization of the memorized face images, which is in line with the sensory-recruitment theories of WM.

## Data Availability Statement

The data used in the statistical analysis can be accessed from the OSF (https://osf.io/9rc4j/). Raw EEG data and scripts used for the presentation of the experimental procedure and data analysis will be shared by the authors per request.

## Ethics Statement

The studies involving human participants were reviewed and approved by Human Ethics Committee of the SWPS University of Social Sciences and Humanities (Warsaw, Poland). The patients/participants provided their written informed consent to participate in this study.

## Author Contributions

MB: conceived the study. NR: collected the data and drafted the manuscript. ŁD and MB: revised the manuscript. All authors designed the study, analyzed the data, and approved the submitted version.

## Conflict of Interest

The authors declare that the research was conducted in the absence of any commercial or financial relationships that could be construed as a potential conflict of interest.

## Publisher’s Note

All claims expressed in this article are solely those of the authors and do not necessarily represent those of their affiliated organizations, or those of the publisher, the editors and the reviewers. Any product that may be evaluated in this article, or claim that may be made by its manufacturer, is not guaranteed or endorsed by the publisher.

## Footnotes

1 Available at http://www.gimp.org/

2
https://osf.io/9rc4j/

3
http://statcheck.io/index.php
